# Combined biological effects of CBCT and therapeutic X-ray dose on chromosomal aberrations of lymphocytes

**DOI:** 10.1186/s13014-024-02504-8

**Published:** 2024-08-14

**Authors:** Ádám Gáldi, Gyöngyi Farkas, Szilvia Gazdag-Hegyesi, Enikő Koszta, Péter Ágoston, Csilla Pesznyák, Tibor Major, Zoltán Takácsi-Nagy, Csaba Polgár, Zsolt Jurányi

**Affiliations:** 1 Centre of Radiotherapy, National Institute of Oncology, Ráth György U. 7-9, Budapest, 1122 Hungary; 2 Department of Radiobiology and Diagnostic Onco-Cytogenetics, Centre of Radiotherapy, National Institute of Oncology, Ráth György U. 7-9, Budapest, 1122 Hungary; 3 Institute of Nuclear Techniques, University of Technology and Economics, Budapest, Hungary; 4 Present Address: Doctoral School of Physical Sciences, University of Technology and Economics, Budapest, Hungary; 5 Department of Oncology, National Institute of Oncology, Semmelweis University, Ráth György U.7-9, Budapest, 1122 Hungary; 6 Doctoral College, Semmelweis University, Budapest, Hungary

**Keywords:** Cone beam computed tomography, CBCT, Chromosome aberrations, IMRT, IGRT, Biodosimetry, In vitro irradiation, Adaptive radiotherapy

## Abstract

**Background and purpose:**

Cone beam computed tomography (CBCT) is routinely used in radiotherapy to localize target volume. The aim of our study was to determine the biological effects of CBCT dose compared to subsequent therapeutic dose by using in vitro chromosome dosimetry.

**Materials and methods:**

Peripheral blood samples from five healthy volunteers were irradiated in two phantoms (water filled in-house made cylindrical, and Pure Image CTDI phantoms) with 6 MV FFF X-ray photons, the dose rate was 800 MU/min and the absorbed doses ranged from 0.5 to 8 Gy. Irradiation was performed with a 6 MV linear accelerator (LINAC) to generate a dose–response calibration curve. In the first part of the investigation, 1–5 CBCT imaging was used, in the second, only 2 Gy doses were delivered with a LINAC, and then, in the third part, a combination of CBCT and 2 Gy irradiation was performed mimicking online adapted radiotherapy treatment. Metaphases were prepared from lymphocyte cultures, using standard cytogenetic techniques, and chromosomal aberrations were evaluated. Estimate doses were calculated from chromosome aberrations using dose–response curves.

**Results:**

Samples exposed to X-ray from CBCT imaging prior to treatment exhibited higher chromosomal aberrations and Estimate dose than the 2 Gy therapeutic (real) dose, and the magnitude of the increase depended on the number of CBCTs: 1–5 CBCT corresponded to 0.04–0.92 Gy, 1 CBCT + 2 Gy to 2.32 Gy, and 5 CBCTs + 2 Gy to 3.5 Gy.

**Conclusion:**

The estimated dose based on chromosomal aberrations is 24.8% higher than the physical dose, for the combination of 3 CBCTs and the therapeutic 2 Gy dose, which should be taken into account when calculating the total therapeutic dose that could increase the risk of a second cancer. The clinical implications of the combined radiation effect may require further investigation.

## Introduction

Nowadays, in the highly conformal irradiation techniques available, such as intensity-modulated radiotherapy (IMRT), the use of some kind of imaging devices is mandatory to ensure that the patient is in the intended treatment position before the treatment is started. This procedure is called Image-Guided Radiotherapy (IGRT). Cone Beam Computed Tomography (CBCT) is an essential radiographic tool in the modern radiotherapy era. CBCT can operate with megavoltage (MV) and kilovoltage (kV) energy. kV-CBCT is a commonly used imaging system in radiotherapy, because of its good image quality and high spatial resolution. This device is always integrated into a linear accelerator (LINAC), making the CBCT a very simple, fast, and precise tool to position the patient on the treatment couch.

The frequency of CBCT depends on the IGRT protocol used [1]. Online or offline protocols can be used, depending on the protocol of the institute. At our institute, we routinely use the extended no action level (e-NAL) offline protocol. With this procedure, we use CBCTs in the first three fractions, and then calculate the mean systematic error and adjust the original isocenter with this error from the 4th fractions onwards. After the adjusted position, patient set-up is verified once a week with kV-CBCT. With this protocol, on average, 10 kV-CBCT imaging is performed for the patients suffering any type of cancer during the full course of treatment.

Detecting anatomical differences and changing patient position are only one of the options of how to use a kV-CBCT. With near-diagnostic image quality, CBCT can also be used for online adaptive radiotherapy (oART). With oART, anatomical changes in the irradiated region are taken into account on a day-by-day basis, and a new irradiation plan is created for the patient. This technique involves daily CBCT scans.

At our institute, an Ethos mono energy LINAC (Varian, Palo Alto, CA, USA) was installed for online adaptive radiotherapy in June 2022. We started the adaptive radiotherapy treatments for patients with non-muscle-invasive bladder cancer. Since then mainly patients suffering cancer in pelvis are treated with oART. During the adaptation and treatment process, patients are scanned with kV-CBCT three times at each fraction on the first three consecutive treatment days. After the third fraction, patients are scanned two times per fraction. This method produces an average of 70 kV-CBCT acquisitions per patient. This number is seven times more than in the conventional treatment method. The measurement of dose from imaging has historically been ignored because of the cost–benefit of the IGRT technique. Alaei et al. summarized that the high-frequency application of modern IGRT techniques is associated with high doses [2]. The LET (Linear Energy Transfer) value is higher for low-energy beam than for the therapeutic beam, so the biologically effective dose (BED) could be higher than assumed [3].

It is known that radiation-induced chromosomal aberration (CA) is a well-measurable in vitro parameter. In a previous study, we compared the biological effects of different irradiation modalities by in vitro chromosome dosimetry. We found significant differences in the dose–effect curves of different photon energies. The radiobiological effect of the Flattering Filter Free (FFF) mode was higher than that of the Flattering Filter (FF) mode. We also showed that lower energy (6MV vs. 10 MV) induced more dicentric plus ring aberrations [4].

A number of studies on CA after low-energy radiation have been published in the literature, there are some on dental kV-CBCT, but only few on kV-CBCT in high frequency, combined with therapeutic radiation energy. Abe et al. showed that dicentric formation was significantly increased in peripheral blood lymphocytes after a single CT scan (mean: 24.24 mSv) [5].

However Qiu et al. [6] have published that patients treated with IMRT or RapidArc in the pelvic region are not at additional risk when using kV-CBCT-based IGRT [6].

Kench et al. found that irradiation of cancerous and normal human cell lines with CBCT reduced mean cancer cell survival as predicted [7].

Sakane et al. measured the biological effects of low-dose (LD) chest CT on chromosomal DNA. Peripheral blood samples were taken from 209 participants before and 15 min after CT scans. The number of CAs after CT was 7.2 and 9.7 per 1000 metaphases in the LD CT (1.5 mSv) and Standard (SD) CT (5 mSv) groups, respectively. The number of CAs was significantly increased after SD CT (*P* = 0.003). [8]. It can be seen, that radiation with a high LET value has a considerable biological effect. This has not been investigated when a large number of imaging test are performed. It is important to examine not only the physical but also the biological effects of kV imagers. In vitro and in vivo studies would be necessary to study the biological effect of radiation. Prospective studies will also be necessary, where process of lymphocyte recirculation be taken into account.

The aim of our study was to assess, through in vitro chromosome dosimetry, the biological effects of the combination of high-energy therapeutic beams with frequent low-energy imaging.

## Materials and methods

This study was approved by the Hungarian Ethical Committee, ETT TUKEB (23,546–3/201) (*23,546–3/2017/EKU*) and was conducted following the principles of the Helsinki Declaration. The five subjects were informed about the aim of the study and gave their written informed consent.

### Blood sample

Venous blood samples were collected from five healthy non-smoking volunteers (three females, two males (age 37.6 ± 12.3 years) in Li-heparinized vacutainers. The blood test was preceded by a routine occupational medical examination. They filled a questionnaire specifying, date of birth, smoking status, dietary habits, alcohol consumption, exposure to diagnostic X-ray, use of therapeutic drugs. All were healthy and non-smokers. The maximum blood volume obtained was 20 ml per volunteer. Spontaneous chromosome aberration baseline values were determined before starting the experiments. We chose healthy subjects for our study because we wanted to measure the absolute effect of radiation on the human chromosome as accurately as possible. However, in a healthy population the age, smoking, long-term medication, drugs, alcohol, toxic chemicals, some viruses and bacteria could cause baseline shift in the number of total aberration.

If we had used blood from cancer patients, this may have affected the aberrations we wanted to measure.

### Phantoms for measurements

**Fig. 1:**
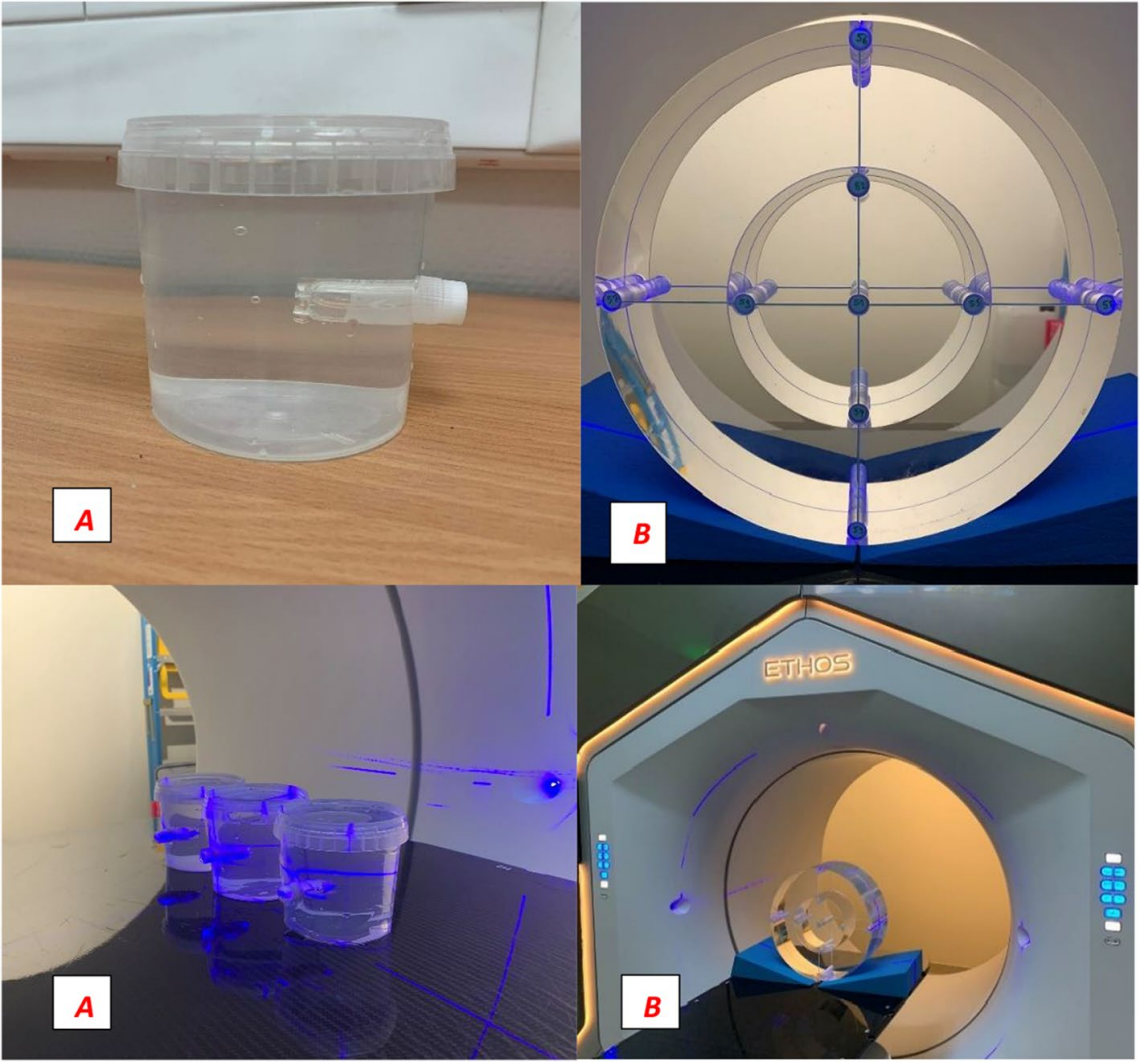
Small cylindrical water-filled phantom with cryotube (**A**) and PMMA CTDI phantom with body cylinder and nine cryotubes (**B**)

In our study, we used measurement setups similar to the clinical routine of oART. We used two different phantoms for the measurements (Fig. 1). The first phantom was an in-house made small box phantom filled with clean water, cylindrical in shape (small cylindrical phantom), with a volume of approximately 380 cm^3^, and 80 mm in diameter and 80 mm in height. The phantom contained only one hole for a 2 ml cryotube, 11 mm in diameter. The second phantom was a Pure Imaging CTDI PMMA phantom (Pure Imaging Phantoms, Spring Court, Farnham Royal,UK) [9] with a body cylinder, approximately 11,965 cm^3^ in volume, 320 mm in diameter and 145 mm in length. This phantom contained nine rods, which could be exchanged for perfectly fitting cryotubes. At our institute, oART treatments started in the pelvic region, so the choice of this body phantom was appropriate.

### CT simulation

To reproduce a realistic treatment situation, we prepared CT-based external treatment plans for both phantoms. We used radiotherapy CT protocols depending on the width of the phantom. The used protocols were the pelvis for CTDI phantom with 120 kVp voltage and 210 mAs current and lung for water-filled with 120 kVp voltage and 35 mAs current. Water-filled cryotubes (2 ml) were inserted into the phantoms before CT imaging. CT images were imported into the Ethos Treatment Planning System 2.1, (TPS) (Varian, Palo Alto, CA, USA).

### Irradiation planning

The cryotube was considered as the gross tumor volume (GTV), but we expanded it by a uniform margin of 1 cm to create the planning target volume (PTV). This volume was used to prescribe the dose. With this PTV, we were able to account for possible phantom shifts and also for the scattered dose from phantom materials. The small cylindrical phantom had only one readout point in the center of the PTV. The CTDI phantom contained nine holes, so we could use all of them for readout points. The PTV volume was placed in the center of the phantom and the volume of the other eight cryotubes was outlined and then used to read the dose.

**Fig. 2:**
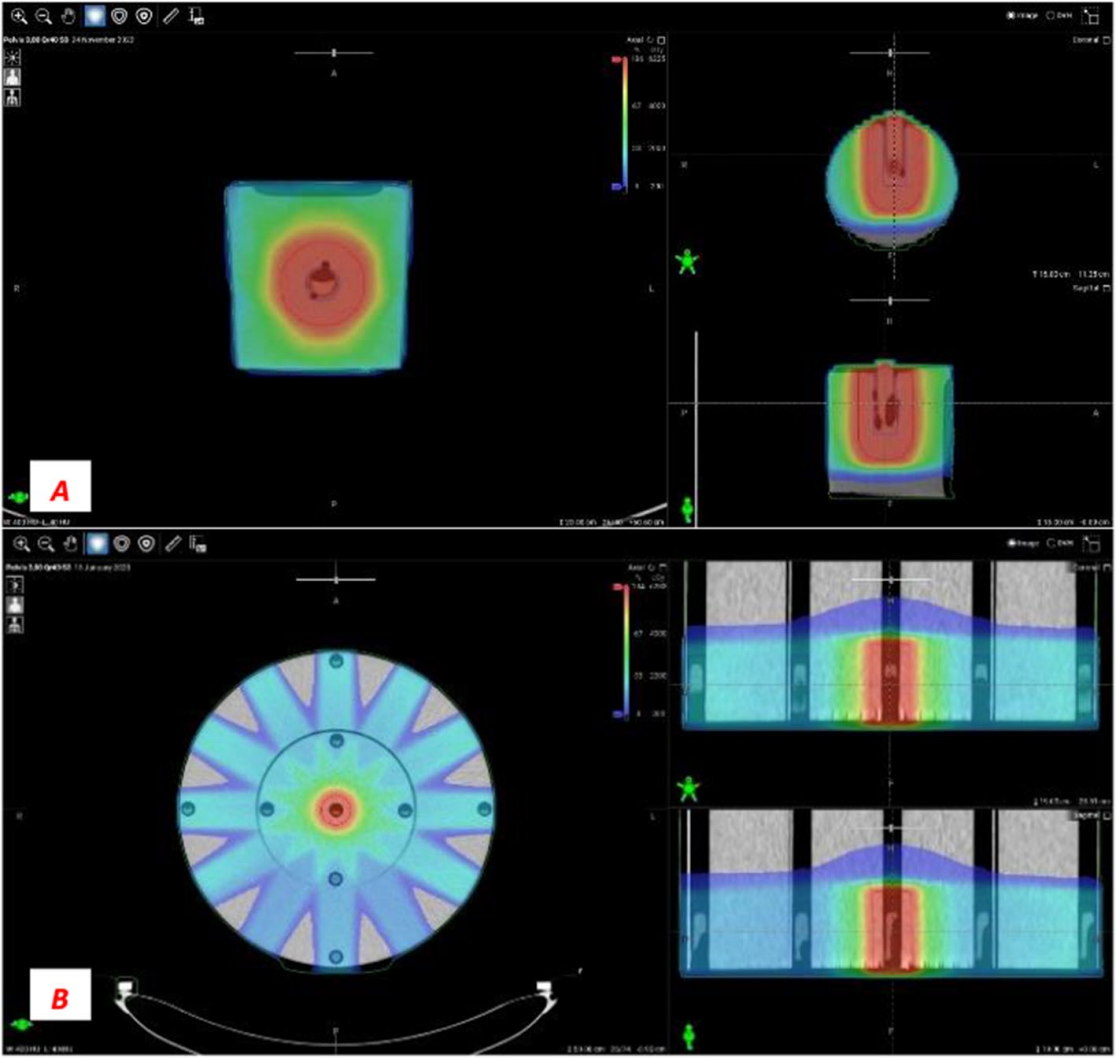
Small cylindrical (**A**) with one, and CTDI phantom (**B**) with the nine read-out volumes (small circles), and dose distributions in three views

There are several options to create a plan for this phantom. It is possible to add only one direct field to the phantom and normalize the dose to the cryotube [10]. In our investigation, “real” treatment plans were created, which means that intensity-modulated radiotherapy plans (IMRT) using the sliding window technique (SW) were used. This was an important criterion in our investigation because IMRT SW plans were also generated for oART. We used 12 equidistant fields with 10-degree collimator rotation. The isocenter was placed in the middle of the central cryotube. We used 6 MV FFF beam energy with a maximum dose of 800 MU/min (therapeutic ray) (Fig. 2).

We kept the 100% average dose requirement for GTV volume, and 100% of the PTV was to be covered by 95% of the prescribed dose, ensuring accurate dose coverage. The monitor units delivered varied depending on the dose prescribed per tube, 268 MU at 0.5 Gy, 414 MU at 1 Gy, 761 MU at 2 Gy, 1543 MU at 4 Gy, 2575 MU at 6 Gy, and 3416 MU at 8 Gy, respectively. The dose volume histogram (DVH) integrated into the planning system was used for post-processing of the dose data. With this tool, the data could be analyzed visually and numerically in the entire study volume.

### Irradiation of blood samples

Reproducing a real-life situation, we set up the phantom using the same procedure as we set up patients in clinical practice. In the Ethos system, the first step is to position the phantom using external lasers. The phantom on the couch is then automatically shifted into the isocenter by the LINAC using precalculated translation values.

We placed a cryotube in the small cylindrical phantom with 2 ml of blood in the central hole. In the CTDI phantom, we placed one cryotube with the blood sample in the central position of the phantom, the other eight holes were filled with water-filled cryotubes as in planning CT imaging.

The same type of imaging protocol was used for the both phantoms, namely pelvis large protocol, for exact comparison. The high voltage was 140 kV, and the exposure was 1068 mAs, the calculated CTDIvol based on TPS (volumetric Computer Tomography Dose Index) was 0.038 Gy, the DLP (Dose Length Product) was 0.852 Gy*cm, and the range was 19.4 cm. However, the actual physical dose may differ from that presented by TPS because of the uncertainty of the input data for dose modelling, dose calculation, commissioning heterogeneity changes, CT calibration, which can cause errors of up to 3–5 percent so it is important to know the real biological effect of CBCT [11]. The use of this imaging protocol was essential because online adaptive therapy requires high image quality and good spatial resolution, independently of the irradiated region.

The use of an imaging guidance is mandatory before starting a treatment with Ethos. Therefore, when we used the therapeutic beams for the calibration curves, we had to scan the phantom with water-filled cryotube instead of a blood sample. After imaging and registration, the tube was replaced with the blood-filled cryotube.

### Separate irradiated blood sample measurement

We investigated several dose delivery setups to measure clinically relevant chromosome aberrations from the kV-CBCT and the therapeutic beams. We generated calibration curves with predefined dose steps (0.5, 1, 2, 4, 6, 8 Gy) with both phantoms. Henceforth, the prescribed dose of 2 Gy was clinically relevant for us. We chose this therapeutic fraction dose because it is the most commonly used in both conventional and adaptive radiotherapy. Afterwards, we irradiated blood samples with 1 to 5 consecutive CBCT. Measurements were performed by placing blood samples in each hole of the CTDI phantom one by one and irradiating with 3 or 5 CBCT to evaluate the peripheral aberration.

### Co-irradiated blood sample measurement

We then mixed the CBCT frequency and therapeutic beam frequencies to evaluate real situations. The dose from the therapeutic beams was the same in every situation (2 Gy), the number of the CBCTs were 1, 2, 3, 4 and 5. The most important configuration investigated was the 3 CBCTs with 2 Gy therapeutic beam because this was our online adaptation schema. We also paid attention to the sequence of delivery. We performed 2 CBCTs first, followed by therapeutic beams, and finished with one CBCT. We performed measurement in the center of the CTDI phantom with only 3 CBCTs and only 2 Gy therapeutic dose to investigate whether the effects of the two types of radiation could be biologically combined and what additive biological effects the combined radiation had.

### Lymphocyte cultures

Blood samples were irradiated at dose rates ranging from 1 to 800 MU/min and 6 MV FFF energy with a dose of 0.5–8 Gy. Culture and chromosome preparation were performed using standard cytogenetic techniques after exposure: 0.8 ml of blood was added to 9 ml of RPMI-1640 culture medium containing 15% bovine serum albumin and penicillin/streptomycin (0.5 ml/L). Cell proliferation was induced with phytohaemagglutinin M (0.2%). Incubation time was 52 h at 37 °C. Lymphocyte proliferation was inhibited with 0.1 μg/ml Colcemid (Gibco) during the last 2 h of culture. Cell cultures were then centrifuged, treated with a hypotonic solution of 0.075 M KCl at 37°Cfor 15 min and fixed with a 3:1 solution of cold methanol-acetic acid. After several washes in fresh fixative, the cells were resuspended in a small volume (0.5 ml) of fresh fixative, then this suspension was dropped on glass slides, dried and stained with 3% Giemsa.

### Study of chromosomal aberrations

Between 100 and 200 metaphases were analyzed per experimental points in manual mode with a light microscope at × 1500 magnification. Chromosome analysis was performed at the first cell division. Only clear oval metaphase cells were counted. All aberration types were recorded. On the basis of structural differences, chromatid-type breaks (chromatid break, exchange) and chromosomal-type breaks and rearrangements (fragments, dicentrics, rings, translocations) were identified. One dicentric or ring and one acentric fragment observed in the examined cell were counted as one chromosome aberration (CA). One tricentric chromosome was counted as two dicentric equivalents. Excess fragments were not distinguished as terminal or interstitial deletions according to the position of the chromatin loss. Acentric fragments without dicentric or ring aberrations were counted as excess fragments. The evaluation was performed according to ICPEMC requirements [12]. Slides were coded and metaphases were analyzed by three well-trained scorers. For scoring, 100–300 complete metaphase cells were evaluated per dose point per donor in all conditions. A total of 158 samples were irradiated and a minimum of 31,600 metaphases were evaluated.

### Statistical analysis

The yields of aberrant cells and aberrations (Y) were expressed per 100 cells scored. Standard errors (SE) for the mean aberration yield were calculated from the dispersion (σ^2^) of aberration among cell distributions. At each experimental point, aberration among cell distributions was checked for consistency with Poisson model using the variance-to-mean ratio (σ^2^ / Y) and Papworth’s u-test [13]. The dose response was fitted to a linear- quadratic model using the iteratively reweighted least squares method. Student's t-test was used for statistical analyses. Significant differences were determined at 95% confidence interval, with a P value of < 0.05 considered as the limit of significance. Dose response was fitted using CABAS-2 (Chromosomal Aberration Calculation) software [14] and GraphPad Prism 5 was used for calculations and data presentation [15]. We did not perform low-dose calibration curves because we wanted to evaluate the summarized aberrations from low-energy and high-energy radiation relatively. For this consideration, we fitted the low-dose energy values in the high-energy calibration curves. In case when a low energy calibration curve was used, it would not had been possible to evaluate the effect of the therapeutic dose, since it is known that it has a larger impact. We know that the most precise procedure would be to evaluate the low energy CBCT dose separately using a low energy calibration curve and the therapeutic dose separately using a high energy calibration curve, but in this case we would not be able to measure the combined effect of the two types of radiation. In addition, in a prospective human study, we would not be able to take blood samples after CBCT radiation and before the start of treatment, so the most feasible approach would be to use a high-energy calibration curve, as we done.

## Results

**Fig. 3:**
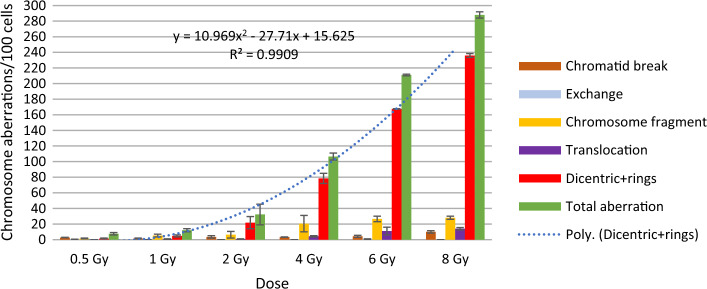
Frequency of chromosomal aberrations of lymphocytes in the blood from donors irradiated with 6MV FFF photon beam at 0.5–8 Gy (average of two measurements) in the small cylindrical phantom

**Fig. 4:**
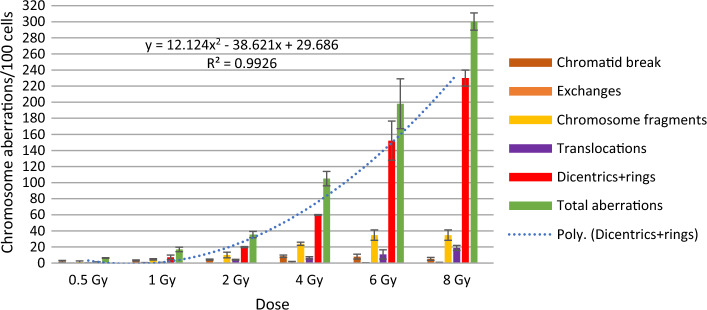
Frequency of chromosomal aberrations of lymphocytes in the blood from donors irradiated with 6 MV FFF photon beam at 0.5–8 Gy (average of 3 measurements) in the CTDI phantom

Figures 3 and 4 show the frequency of different chromosomal aberrations for the therapeutic calibration curve, averaged over five measurements of blood lymphocytes from donors. We found no significant differences in the frequency of aberrations between the two phantoms. The frequency of different aberrations increased with dose. Chromatid type aberrations were very low, and were not typical after irradiation. Dicentric plus rings were the most frequent and specific aberrations upon irradiation. Their number increased quadratically with the dose. Dicentrics plus rings accounted for 75.9–81.9% of all aberrations at 8 Gy. Of radiation specific dicentric and ring aberrations, dicentric chromosomes were 6–10 times more frequent than rings. Comparing the incidence of irradiation, we found that specific ring aberrations in this study (in small cylindrical and in CTDI phantom) were higher than in our previous work at 6–10 MV and 6–10 MV FFF irradiation [4]. In that experiment, a maximum of 0–2 rings were observed at 0.5–2 Gy. In contrast, our present results show that at 3 CBCTs + 2 Gy, 2–5 CBCTs resulted in 1–4, 2–6 rings aberrations. The number of translocations and chromosome fragments also increased quadratically, but by an order of magnitude less than dicentrics.

**Fig. 5:**
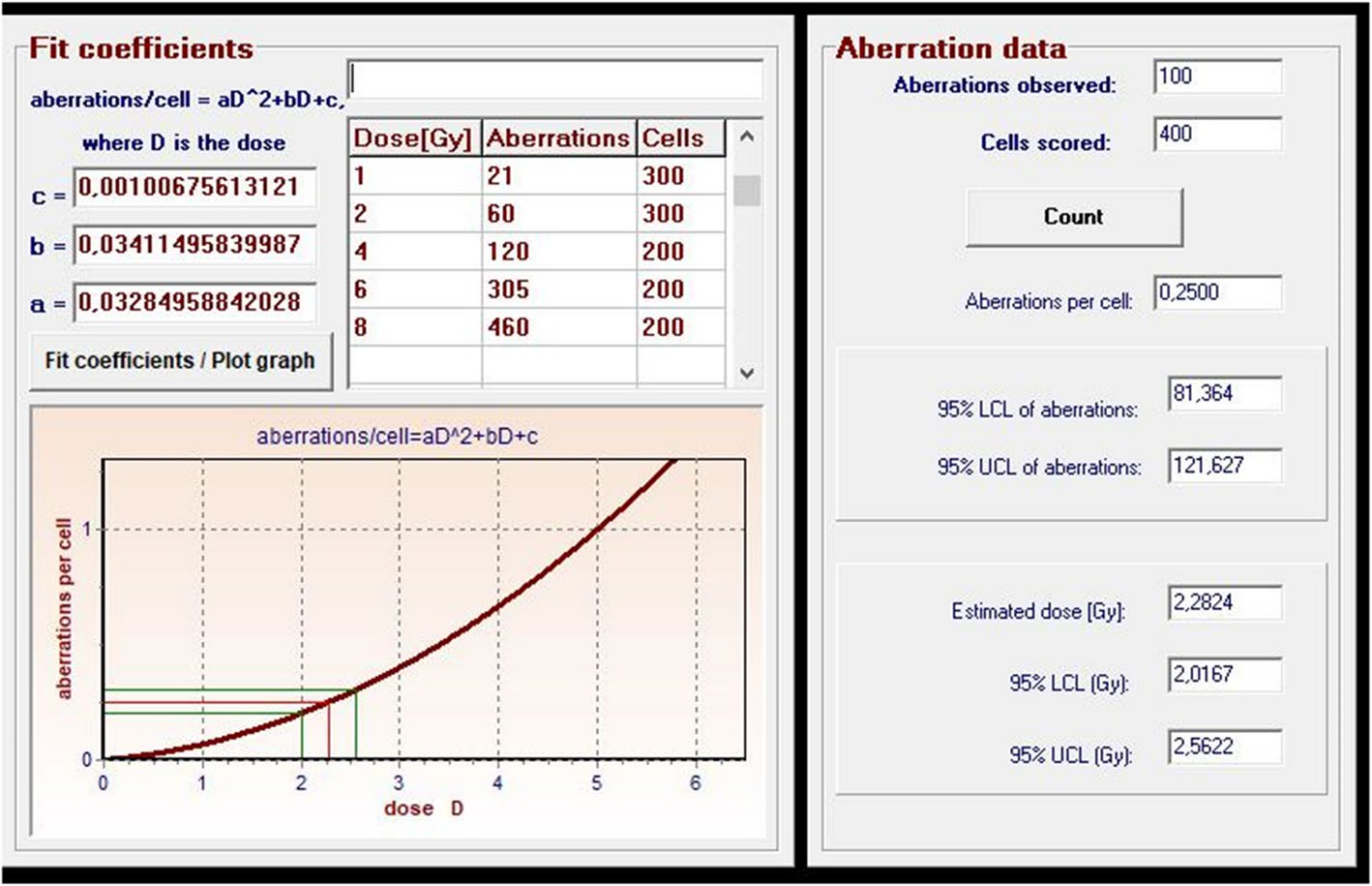
Dose- response calibration curves for dicentrics and rings induced by irradiation with ETHOS X-ray using CTDI phantom

Figure 5 shows the dose–response calibration curve for dicentric and ring chromosomes induced by irradiation with therapeutic beams.

Dose–response was fitted using CABAS-2 (Chromosomal Aberration Calculation) software [16]. The formula of the curve is* Y* = *c* + *αD* + *βD*^2^, where *Y* is the number of dicentric and ring chromosomes/number of metaphase spreads scored, *D* is the radiation dose, *c* is the background level, and *α* is the linear and *β* is the quadratic coefficient: **Y = 0.0009 + 0.041D + 0.036 D**^**2**^ and **Y = 0.0009 + 0.026D + 0.035 D**^**2**^**.** The first formula is the equation obtained from measurements with the small cylindrical phantom and the second one with the CTDI phantom. We compared the parameters of the equation with literature data and data obtained previously[4, 17]. The values of *β* were similar, because we use same energy and similar dose rate (*β:0.036 in our current study, and 0.044 in the referred literature*).

### The effect of CBCT scan on chromosomal aberrations

**Fig. 6:**
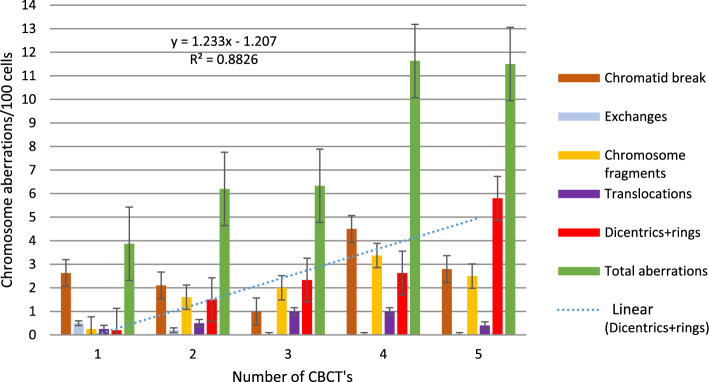
Frequency of chromosome aberrations of lymphocytes in the blood from two donors irradiated with140kV CBCT beam, with 1–5 CBCT scans, in the small cylindrical phantom

**Fig. 7:**
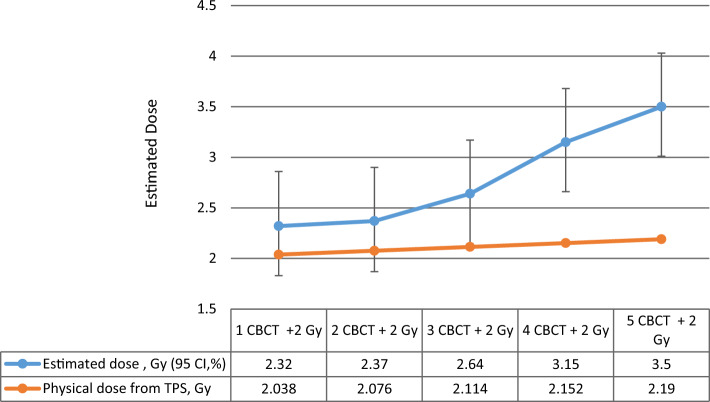
Estimated doses (Gy) of chromosome aberration (dicentrics + rings) values based on calibration curve (Fig. 1) by CABAS, using the small cylindrical phantom

In the following series of our experiments, the blood samples were first scanned with 1–5 CBCTs, and then in another experiment, the samples were irradiated with 1–5 CBCTs + 2 Gy, and chromosomal aberrations were compared (Figs. 6, 7 Dicentric and ring chromosomal aberrations increased linearly with the number of CBCTs. The value of dicentrics and ring increased significantly with therapeutic dose. Using the dose curve (Fig. 5), we were able to estimate the dose values corresponding to dicentric and ring chromosomes (Fig. 7). Although 1 CBCT had no significant biological effect, 3–5 CBCTs corresponded to a dose of 0.3–0.5 Gy. Depending on the number of the CBCT + 2 Gy, the estimated biological dose was higher than the physical dose by 14% (1–2 CBCTs), 24.8% (3 CBCTs), 46.4% (4 CBCTs) and 59.8% (5 CBCTs).

For 2–3 CBCTs and 4–5 CBCTs, the total number of aberrations was similar, but the quality was different. As the number of chromatid breaks decreased, the number of dicentrics increased with increasing CBCT dose (Fig. 6). There was a significant difference between 3 and 5 CT, in dicentrics and rings (*p* = 0.006), in total aberrations (*p* = 0.007), in aberrant cells (*p* = 0.004) with CTDI phantom. (N = 7 5CT, N = 10 3 CT). Significant differences: in chromatid breaks (*p* = 0.029) between 3 and 5 CT, in chromosome fragments 1 versus 2 CT (*p* < 0.0001), 1 versus 3 CT (*p* = 0.004), 1 versus 4 CT (*p* = 0.046), 1 versus 5 CT (*p* = 0.001), in dicentrics and rings 1 versus 2 CT (*p* = 0.017) 1 versus 4 CT (*p* < 0.0001), 1 versus 5 CT (*p* < 0.0001), 2 versus 5 CT (*p* < 0.0001), 3 vs. 5 CT (*p* = 0.036), in total aberrations 1 versus 4 CT (*p* = 0.029), 1 versus 5 CT (*p* < 0.0001), 2 versus 5 CT (*p* < 0.0001), 3 versus 5 CT (*p* = 0.043), in aberrant cells 1 versus 4 CT (*p* = 0.024), 1 versus 5 CT (*p* < 0.0001), 2 versus 5 CT (*p* < 0.0001), 3 versus 5 CT (*p* = 0.043).

### Comparison of radiation for 3 and 5 CBCTs

**Fig. 8:**
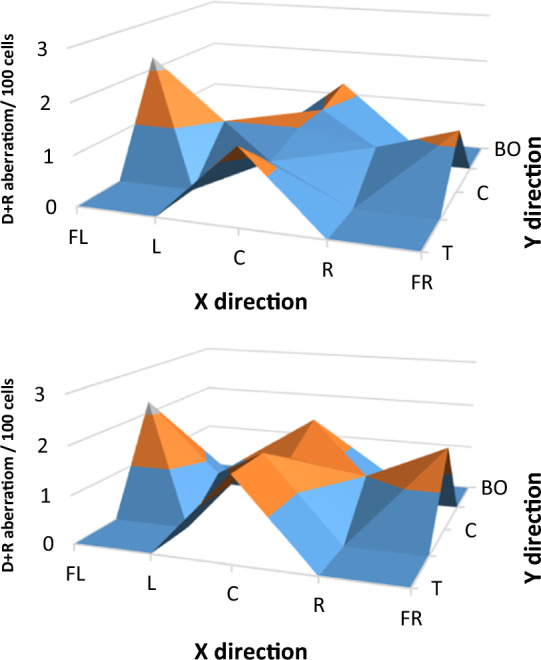
Frequency of average dicentric and ring chromosomal aberrations of lymphocytes in the blood from donors after three and five CBCT scan. Samples were irradiated in different positions in the CTDI phantom. Labels: T-Top, U-UP, C-Center, BE-Below, BO-Bottom, FL-Far left, L-Left, R-Right, FR-Far Right

Blood samples were inserted into the CTDI phantom at different positions (nine positions, see Fig. 1) and the number of chromosomal aberrations was determined after 3 or 5 CBCTs, representing a clinical situation (3 CBCTs), and worst case scenario (5 CBCTs). The average of seven measurements of chromosomal aberrations differed slightly but only in chromatid breaks was significant difference between 3 and 5 CT (*p* = 0.026) With one or two exceptions, the aberrations of 5 CBCTs were higher than those of 3 CBCTs. Chromatid breaks were between 1 and 4, and exchanges were very low, zero at most positions. Dicentric and ring aberrations were more important, between 0.75 and 2.25. Total aberrations occurred in 3–7 out of 100 cells, and almost all had translocations, which were stabile aberrations. However, the clinical significance of the aberrations seems to be negligible (Fig. 8). A small difference can be observed at the far left measurement point, this may be due to the half bowtie CBCT beam profile.

### Aberration of mixed energy beam in CTDI phantom

**Fig. 9:**
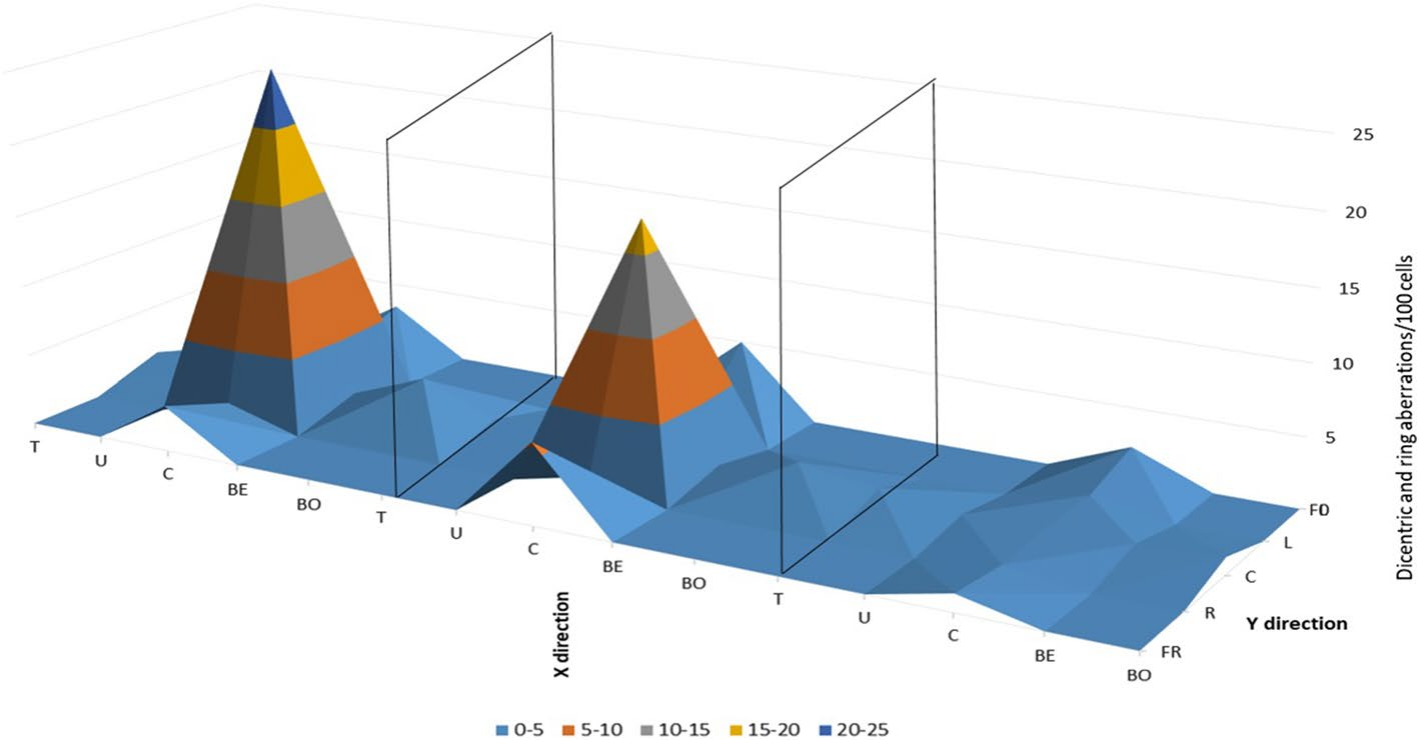
Summarized visualization of CTDI phantom measurement in nine readout points, in three irradiation settings, 3CBCT + 2 Gy (left), 2 Gy only (middle), and 3 CBCTs only (right). T-Top, U-UP, C-Center, BE-Below, BO-Bottom, FL-Far left, L-Left, R-Right, FR-Far Right Figure 10.

In the next experiment series, samples placed in the holes of the phantom were irradiated only in the center, but all types of chromosomal aberrations were examined in all samples and in all readout points. The central sample was irradiated with only 2 Gy or 2 CBCTs plus 2 Gy and plus 1CBCT or only 3 CBCTs (Fig. 9). Dicentric and ring aberrations (*p* = 0.007), total aberrations (*p* = 0.001) and aberrant cells (*p* = 0.008) were significantly higher with CBCT dose than without and we found that the estimated doses of different types of radiation (kV and MV X-rays) could not be quantified separately.

Aberrations were also found in peripheral samples not directly irradiated. On average, there were 2.12 dicentrics and rings, 6.68 total aberrations, 6.28 aberrant cells/100 cells in the sample for 2 CBCTs, + 2 Gy + 1CBCT, and 1.34 dicentrics and rings, 4.65 total aberrations and 4.65 aberrant cells/100 cells for 2 Gy.

**Fig. 10:**
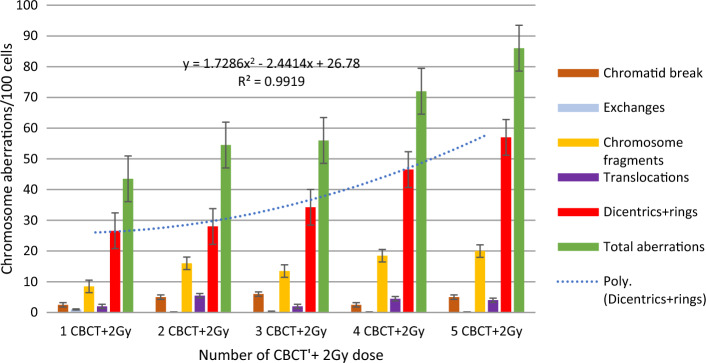
Chromosome aberrations after using different numbers of CBCT with a therapeutic dose of 2 Gy, measured in the CTDI phantom in the center read outpoint

We performed measurements in the CTDI phantom when we combined the frequency of the CBCTs and therapeutic 2 Gy and found that the beam-specific dicentric and ring and total aberrations increased exponentially when high-LET radiation was combined with low LET (Fig. 10).

## Discussion

The biological effect of adding a CBCT dose to a therapeutic dose is poorly understood. In our work, we studied the biological effect of CBCT in vitro in comparison with a therapeutic dose of 2 Gy. For both types of phantoms (small cylindrical and CTDI), a dose response-curve was taken in the range of 0.5–8 Gy. With the help of these dose- response curves, based on the chromosomal aberrations, we estimated the biological doses belonging to 1–5 CBCTs, which was 0.3–0.5 Gy. There was a difference in the estimated doses between the two experimental setups.

We compared the biological effects of 3 and 5 CBCTs using blood samples placed at different positions in the phantom. We compared the effects of 2 Gy or 3 CBCTs plus 2 Gy on 2 samples each, which were placed in the center of the phantom and in both cases also in the peripheral part of the phantom. There was also a difference of 0.3 Gy between samples with and without CBCT. Comparing the summarized therapeutic doses from the TPS and imaging doses written out by LINAC console, we found that they did not correlate with the measured doses. As the frequency of the images increased in parallel with the same therapeutic dose, the differences between the measured doses and summarized doses from TPS and LINAC console were significant (Fig. 7).

The estimated dose based on chromosomal aberrations is higher by 14% at 1–2 CBCTs than the physical dose, by 24.8% at 3 CBCTs, by 46.4% at 4 CBCTs and by 59.8% (5 CBCTs).

We cannot compare our results with similar in vitro tests on lymphocytes. There are some studies on cancer cell lines. Kench et al. found that CBCT dose prior to a therapy dose reduced cell survival more than predicted, corresponding to more than 5% of the therapeutic dose [7]. In another study, primary human fibroblast cells from the lung were irradiated with a single CBCT (16 mGy). The yield of DNA double-strand breaks (gamma-H2AX foci) in the sample increased significantly compared to control group [18]. Dicentric chromosomes in peripheral blood lymphocytes could well be used for detection and dose assessment of human radiation exposure [19]. Peripheral blood lymphocytes were collected from 10 patients before and after CT scans to access effects of low-dose ionizing radiation on chromosomes. Dicentric and ring formation increased significantly after a single CT scan in all patients [20]. Abe et al. also investigated the cumulative number of chromosome aberrations induced by three consecutive CT scans in eight patients [5]. A cumulative increase in the frequency of dicentrics and translocation formation after three consecutive CT scans was observed in the eight patients studied, the chromosomes of the patients may have been affected by ageing, treatment of their disease and smoking.

Image-guided radiotherapy is an essential tool in modern intensity-modulated radiation therapy, and results in higher dose conformity and decreased dose to the surrounding tissue compared to the conventional 3D conformal radiotherapy. The most complex radiotherapy treatment is oART; however, this treatment modality requires the intensive use of CBCT Therefore, the risk of complications and the dose to normal tissues can be higher than estimated.However, significant doses can accumulate out-of-field due to photon scattering with changed energy spectrum what is already known, but it may have impact of the cellular response in these regions and these topics under investigation in many centre.. [21–24].

It seems that the assessment of physical or estimated dose based on chromosomal aberrations of CBCT alone is not an appropriate method to evaluate the biological effects of radiotherapy. There are big differences between the physical and estimated doses when therapeutic and CBCT doses are investigated together. It should to be noted, however, that in oART, these two types of irradiation are applied together, and their combined effect may have clinical implications that require further investigation.

## Limitations

To design our study, we planned to recruit 5 healthy people. The number of volunteers included may be a limitation in our study, although the number of samples from volunteers has adequate statistics in our opinion. Interpreting the results, a limitation is that we tested blood from healthy people while concluding for cancer patients. In addition, we would not be able to take blood from the patient on the treatment table after performing the CBCT, so we chose this approach for our study. However, we detected very small changes in the study group, and these changes would be biased by genetic changes in patients having cancer. We are planning to open prospective human studies after our results have been validated. As a limitation, we used a calibration curve of high energy irradiation to calculate the estimated dose, but in our study we wanted to investigate the combined effect of low and high energy irradiations, using the curve of high energy irradiation, which has a greater impact.

## Conclusion

The biological effect of CBCT is much greater than what can be expected from the physical dose. This effect should be taken into account when calculating the total dose for the treatment of patients. Furthermore, it should be considered at patient selection for oART. Also, prospective human clinical studies comparing treatments with or without CBCT and risk analysis for the development of a second tumor would be needed. To our knowledge, this is the first study to investigate the biological effect of CBCT imaging in combination with therapeutic dose.

## Data Availability

No datasets were generated or analysed during the current study.

## Abbreviations

BED: Biologically effective dose
CA: Chromosomal aberration
CBCT: Cone beam computed tomography
CT: Computer tomography
CTDIvol: Volumetric computer tomography dose index
DLP: Dose length product
e-NAL: Extended no action level
FFF: Flattering filter free
GTV: Gross tumor volume
IGRT: Image-guided radiotherapy
IMRT: Intensity-modulated radiotherapy
kV: Kilovoltage
LET: Linear energy transfer
LINAC: Linear accelerator
MV: Megavoltage
oART: Adaptive radiotherapy
PTV: Planning target volume
SW: Sliding window technique
TPS: Treatment planning system
